# The Factors Affecting Rhythm Control for Cryoablation of Atrial Fibrillation in Mitral Valve Surgery

**DOI:** 10.21470/1678-9741-2019-0064

**Published:** 2019

**Authors:** Fevzi Sarper Türker, Mustafa Bilge Erdogan, Ayşe Dogan

**Affiliations:** 1 Department of Cardiovascular Surgery, University of Health Sciences, Elazığ Training and Research Hospital, Elazığ, Turkey.; 2 Department of Cardiovascular Surgery, Bahçeşehir University Medical School, Istanbul, Turkey.; 3 Department of Physiotherapy and Rehabilitation, Bitlis Eren University Health High School, Bitlis, Turkey.

**Keywords:** Mitral Valve, Cryosurgery, Atrial Fibrillation, Cox-Maze III, Heart Atria, Thromboembolism, Echocardiography

## Abstract

**Objective:**

To evaluate the factors impacting on the conversion to sinus rhythm and on the postoperative rhythm findings in the six-month follow-up period of a mitral valve surgery combined with cryoablation Cox-Maze III procedure, in patients with atrial fibrillation.

**Methods:**

In this study, we evaluated 80 patients who underwent structural valve disease surgery in combination with cryoablation. Indications for the surgical procedures were determined in the patients according to the presence of rheumatic or non-rheumatic structural disorders in the mitral valve as evaluated by echocardiography. Cox-Maze III procedure and left atrial appendix closure were applied.

**Results:**

The results of receiver operating characteristics analysis indicated that the rate of conversion to the sinus rhythm was significantly higher in patients with left atrial diameters ≥ 45.5 mm and with ejection fraction (EF) ≥ 48.5%. However, the statistical differences disappeared in the sixth month. Thromboembolic (TE) events were seen only in three patients in the early period and no more TE events occurred in the six-month follow-up period.

**Conclusion:**

The EF and the preoperative left atrial diameter were determined to be the factors impacting on the conversion to sinus rhythm in patients who underwent mitral valve surgery in combination with cryoablation. Mitral valve surgery in combination with ablation for atrial fibrillation does not affect mortality and morbidity in the experienced health centers; however, it remains controversial whether it will provide additional health benefits to the patients compared to those who underwent only mitral valve surgery.

**Table:** 

Abbreviations, acronyms & symbols				
AF	= Atrial fibrillation		MVS	= Mitral valve surgery
AUC	= Area under the curve		PV	= Pulmonary veins
AVR	= Aortic valve replacement		ROC	= Receiver operating characteristics
CABG	= Coronary artery bypass grafting		SD	= Standard deviation
CPB	= Cardiopulmonary bypass		SE	= Standard error
ECG	= Electrocardiogram		SPSS	= Statistical Package for the Social Sciences
EF	= Ejection fraction		SR	= Sinus rhythm
LAD	= Left atrial diameter		TE	= Thromboembolic
MVR	= Mitral valve replacement			

## INTRODUCTION

Atrial fibrillation (AF) is the most common cardiac arrhythmia in the daily clinical practice, leading to increased incidences of thromboembolic (TE) events and mortality^[1]^. The rates of AF in adults were estimated to increase by two folds at each consecutive decade and it has been shown that 70% of the individuals with AF are between the ages of 65 and 80 years^[2]^.

The AF incidence is high in patients in whom mitral valve surgery (MVS) is indicated^[3]^. At the same time, this condition is a factor increasing mortality^[4]^. The cardiology and cardiac surgery societies frequently update the AF treatment guidelines with the results of many clinical and physiological studies^[5]^.

The Cox-Maze III procedure is currently the golden standard in the surgical treatment of AF, achieving success rates > 90%^[6,7]^. Consequently, after the achievement of these success rates, the surgical treatment of AF has been standardized by the evolving modified versions of the maze procedure^[8]^. However, the procedure's way of preventing the mechanism causing AF recurrences has not been clarified yet. The paramount theory to explain the procedure's protective effect against the development of AF proposes that the right and the left atrial conduction blocks are created and macro reentry circuits are prevented^[9]^.

MVS in combination with surgical AF ablation allows patients to remain in the sinus rhythm (SR) in the short and middle-term periods compared to the MVS performed alone; however, no differences were observed when these two groups were compared in terms of the following measures and events including the 30-day mortality, all-cause mortality, pacemaker implantation, stroke, and TE events^[10]^. According to the patients’ records and retrospective analyses, the application of the maze procedure in combination with the MVS does not lead to increased rates of mortality^[11]^.

We evaluated some factors impacting on the conversion to SR in patients with AF who underwent MVS combined with cryoablation.

## METHODS

### Patients

Eighty patients with left-sided valvular heart disease, suffering from medical treatment-resistant AF (6.25% paroxysmal and 93.75% persistent AF), who were treated in the period between January 2013 and November 2016 were included in the study. The patients were retrospectively evaluated using the information in the digital records of the hospital and in the patients’ files. The indications of MVS were determined by means of transthoracic or transesophageal echocardiography in all patients. Coronary angiography was performed preoperatively in all patients over 45 years old. It was determined whether the patients, who were followed up by the cardiologists, had paroxysmal or persistent AF. The patients with comorbid aortic and/or tricuspid valve diseases, as well as those requiring coronary revascularization, were included in the study too. Patients with infective endocarditis or adult congenital heart disease and those with a history of open-heart surgery or invasive procedures for the treatment of AF were excluded. Routine hematological tests and urinalysis, as well as radiological diagnostics, were performed preoperatively in all patients in order to ensure that no contraindications existed to perform the surgical procedures (Table 1).

**Table 1:** Descriptive statistics for the patients' characteristics.

Characteristic	Male	Female
	**Count (percent)**	**Count (percent)**
Number of patients	31 (38.75)	49 (61.25)
	**Mean±SE mean**	**Mean±SE mean**
Age	48±11.13	46±13.31
Preoperative left atrial diameter	48±4.71	47±4.61
Left ventricular ejection fraction (%)	53±6.86	52±6.94
	**Count (percent)**	**Count (percent)**
Paroxysmal AF	2 (2.50)	3(3.75)
History of embolic stroke	1 (1.25)	1(1.25)
Preoperative heart failure	4 (5.00)	7 (8.75)
Coronary artery disease	9 (11.25)	6 (7.50)
Hypertension	14 (17.50)	22 (27.50)
Diabetes mellitus	4 (5.00)	14 (17.50)
Lung disease	8 (10.00)	7 (8.75)

AF=atrial fibrillation; SE=standard error

This study was approved by our hospital's institutional Ethics Committee, which waived the requirement for patient informed consent because of the retrospective nature of the study.

### Surgical Procedures

All patients and their relatives had been informed clearly about the procedure before surgery and written consent forms had been obtained previously. All patients were operated by cardiopulmonary bypass procedure, undergoing standard sternotomy under general anesthesia. MVS and cryoablation with Cox-Maze III procedure were performed in the flaccid heart with cross-clamping.

**Fig. 1:**
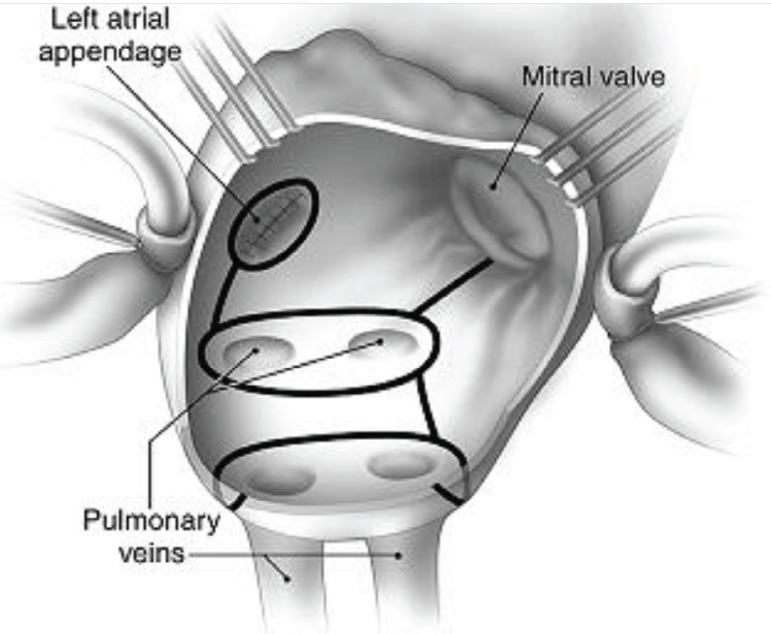
Schematic view of cryoablation of Cox-Maze III procedure.

Induction of general anesthesia was performed in all patients undergoing surgery. A median sternotomy was also performed in all patients. Heparin (350-400 unit/kg) was administered to the patients followed by routine aortic and bicaval cannulation. Membrane oxygenators were used, and moderate systemic hypothermia was applied to perform the cardiopulmonary bypass (CPB) procedure. Myocardial protection was achieved by using antegrade and retrograde mild hypothermic blood cardioplegia (at 32°C), repeated every 20 minutes. The mitral valve procedure was carried out by entering through the Waterstons’ groove or by entering the left atrium from the right atrium via the transseptal way. The transseptal approach was preferred in 25 patients with small left atria or with enlarged left atria extending posteriorly as detected by echocardiography. Primarily, the mitral valve, the orifices of the left atrial auriculae, and the orifices of the pulmonary veins (PV) were examined. Before the mitral valve procedure, the left atrial auriculae were amputated externally and the resulting defect was closed by continuous suturing. Following this procedure, the cryoablation technique was applied to the left atrium; complying with the standard schema (argon-based CryoMaze, CryoFlex Medtronic, Minneapolis, Minnesota, United States of America). After the orifices of the PV were ablated in the posterior walls of the atria, the lines were extended to the mitral valve annulus and left atrial auriculae (Figure 1). Following the ablation, MVS was performed.

Three patients underwent flexible mitral annuloplasty (mitral annuloplasty ring SJM Tailor^TM^) and artificial heart valve replacements were performed in 77 patients (71 mechanical and six biological valvular prostheses from St Jude Medical were used). Left atrial thrombectomy was performed in 19 (23.75%) patients. Aortic valve replacements were performed in eight patients and coronary revascularizations were performed in three patients in combination with mitral valve replacement. Tricuspid valve repairs were performed in 17 patients. Coronary revascularizations were performed in 15 of 80 patients and the mean number of the grafting was 1.4 (Table 2).

**Table 2:** Descriptive statistics for surgical procedures and postoperative results.

	Male	Female
	**Count (percent)**	**Count (percent)**
Number of patients, n	31 (38.75)	49 (61.25)
Mitral ring annuloplasty, n	1 (1.25)	2 (2.50)
MVR + AVR, n	2 (2.50)	6 (7.50)
MVR + AVR + CABG, n	2 (2.50)	1(1.25)
MVR+ CABG, n	8 (10)	4 (5)
Tricuspid valve annuloplasty, n	6 (7.50)	11 (13.75)
	**Mean±SE mean**	**Mean±SE mean**
Cross-clamp time, min	71±5.15	71±6.19
Total CPB time, min	103±11.26	106±11.64
	**Count (percent)**	**Count (percent)**
Left atrial thrombus, n	6 (7.50)	13 (16.25)
Temporary pacemaker, n	4 (5)	10 (12.50)
Mechanical artificial heart valve, n	28 (35)	43 (53.75)
Biological artificial heart valve, n	2 (2.50)	4 (5)
Permanent pacemaker, n	2 (2.50)	4 (5)
Postoperative sinus, n	23 (28.75)	38 (47.50)
Postoperative thromboembolic event, n	1 (1.25)	2 (2.50)
Postoperative permanent thromboembolic event, n		1 (1.25)
	**Mean±SE mean**	**Mean±SE mean**
Duration of hospitalization (days), n	8±3.12	10±5.01

AVR=aortic valve replacement; CABG=coronary artery bypass grafting; CPB=cardiopulmonary bypass; MVR=mitral valve replacement; SE=standard error

### Postoperative Management and Rhythm Assessments

All patients were transferred to the intensive care unit under mechanical ventilation support. Invasive arterial pressure and electrocardiogram (ECG) monitorization were performed in all patients at the intensive care unit and the patients' daily ECGs were recorded. All patients with acceptable levels of bleeding started anticoagulation treatment with enoxaparin and adjunctive warfarin. Enoxaparin treatment stopped when the international normalized ratios were > 2. Some of the patients received an oral beta-blocker (metoprolol) administered in individually adjusted doses for rhythm control. Central TE events occurred in three patients postoperatively. Of these three patients, two were the patients who underwent left atrial thrombectomy. One of these three patients had hemiparesis at the time of discharge; however, two of them were discharged without any neurological sequela.

**Fig. 2:**
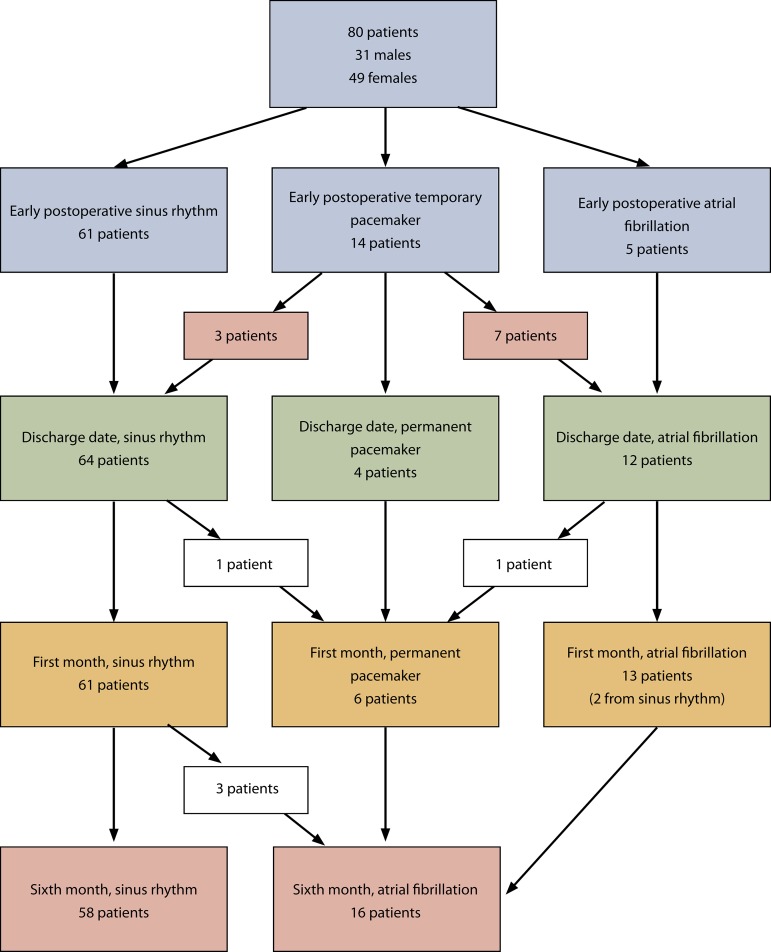
Summary of patients.

ECG recordings were made every six hours in the early postoperative period. In order to evaluate the cardiac rhythm, daily recordings of the ECGs were kept in the inpatient service too. Patients started amiodarone infusion if AF occurred early postoperatively. Conversion to SR did not occur in three patients; therefore, cardioversion was applied to them in the intensive care unit under sedation by midazolam. Eventually, conversion to SR developed in both of these patients (Figure 2).

### Statistical Analysis

As the data determined by the measurements were characterized as more or less with a certain rating, they were subjected to receiver operating characteristics (ROC) analysis and Chi-square independent samples test. The Statistical Package for the Social Sciences (SPSS) software, version 20.0, was used. The results were interpreted according to a *P*-value of < 0.05.

## RESULTS

**Fig. 3:**
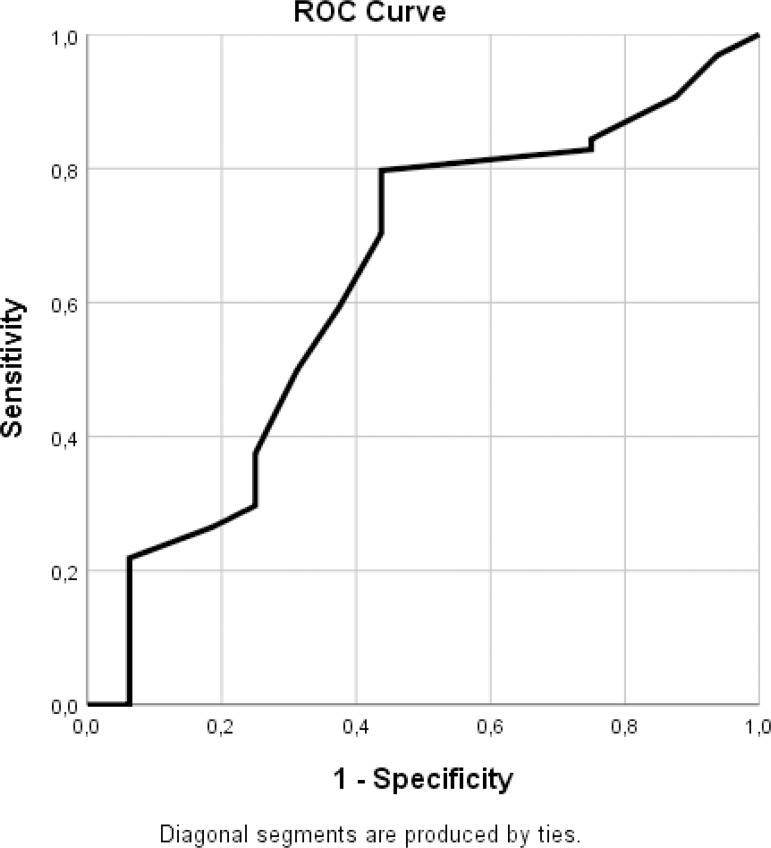
ROC curve for the preoperative LAD value - sinus rhythm on the day of discharge. LAD=left atrial diameter; ROC=receiver operating characteristics.

**Fig. 4:**
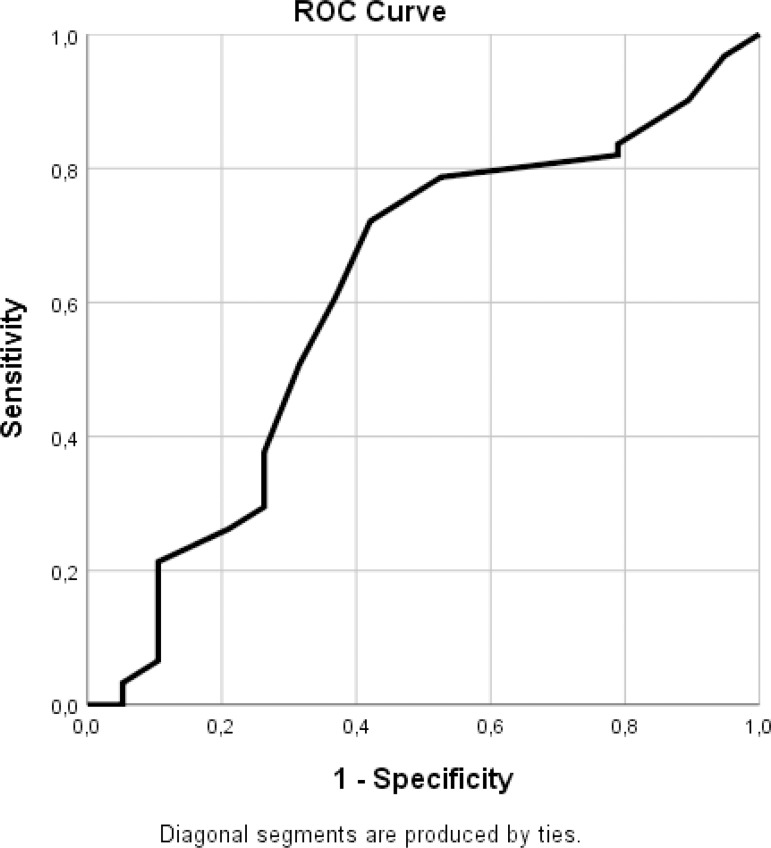
ROC curve for the preoperative LAD values - sinus rhythm in the first month. LAD=left atrial diameter; ROC=receiver operating characteristics.

ROC analysis conducted to determine the cutoff values for the measured values of the preoperative left atrial diameter (LAD) and the ejection fraction (EF) revealed that the area under the curve (AUC) value reached a level over 0.60 (0.633 and 0.613) for the preoperative LAD when the “sinus rhythm on the day of discharge” and the “sinus rhythm in the first month” were used as variables. The point where the sensitivity and specificity values were the highest was accepted as the cutoff value (Figures 3 and 4; Tables 3 and 4). Figure 3 and Table 3 show that a cutoff value of 44.5 mm with a sensitivity of 0.797 and a specificity of 0.562 (1-specificity = 0.438) may be accepted for the preoperative LAD variable when the rhythm on the day of discharge was used. When the “sinus rhythm at the first month” was used as a variable (Figure 4 and Table 4), a cutoff value of 45.5 mm could be accepted (sensitivity = 0.721 and specificity = 0.579) for the preoperative LAD variable.

**Table 3:** Coordinates of the curve for preoperative LAD values - sinus rhythm on the day of discharge.

Coordinates of the curve:		
Positive if greater than or equal to^a^	Sensitivity	1 - Specificity
44.5000 (cutoff value)	0.797	0.438

Test results: Preoperative LAD had at least one tie between the positive actual state group and the negative actual state group.

a The smallest cutoff value is the minimum observed test value minus 1, and the largest cutoff value is the maximum observed test value plus 1. All the other cutoff values are the averages of two consecutively ordered observed test values.

**Table 4:** Coordinates of the curve for preoperative LAD values - sinus rhythm in the first month.

Coordinates of the curve:		
Positive if greater than or equal to^a^	Sensitivity	1 - Specificity
45.5000 (cutoff value)	0.721	0.421

Test results: Preoperative LAD had at least one tie between the positive actual state group and the negative actual state group.

a The smallest cutoff value is the minimum observed test value minus 1, and the largest cutoff value is the maximum observed test value plus 1. All the other cutoff values are the averages of two consecutively ordered observed test values.

Based on the ROC analysis’ results defining the cutoff value for the preoperative LAD, the Chi-square test revealed that the rate of conversion to SR was statistically significantly higher in the group with preoperative LAD values ≥ 46 mm compared to the group with preoperative LAD ≤ 45 mm (Table 5).

**Table 5:** Comparison between two groups of patients (preoperative LAD ≤ 45 mm in one group and ≥ 46 mm in another group).

Preoperative LAD		≤ 45 mm	≥ 46 mm	X^2^	SD	*P*
Postoperative SR	Absent	4	15	0.520	1	0.471
Total	Present	18	43			
		22	58			
Temporary pacemaker Total	Yes	4	10	0.010	1	0.921
	No	18	48			
		22	58			
SR on the day of discharge	Absent	9	7	8.292	1	0.004
Total	Present	13	51			
		22	58			
SR in the first month	Absent	9	10	4.934	1	0.026
	Present	13	48			
		22	58			
SR in the sixth month	Absent	9	13	2.737	1	0.098
Total	Present	13	45			
		22	58			

LAD=left atrial diameter; SD=standard deviation; SR=sinus rhythm

**Fig. 5:**
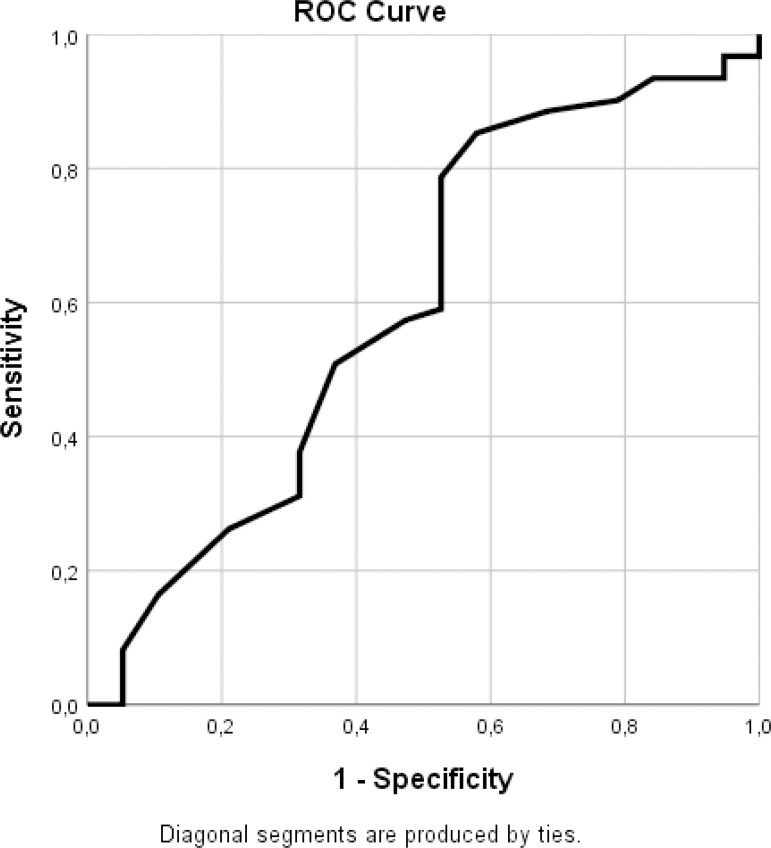
ROC curves for ejection fraction value - postoperative sinus rhythm. ROC=receiver operating characteristics.

The AUC for the EF variable achieved a value of 0.601 when the “postoperative sinus rhythm” variable was used alone (Figure 5 and Table 6). When Figure 5 and Table 6 were evaluated in combination, a cutoff value of 48.5% could be accepted for EF, using the “postoperative sinus rhythm” variable with a sensitivity of 0.787 and specificity of 0.494 (1-specificity = 0.506).

**Table 6:** Coordinates of the curve for ejection fraction (EF) values - postoperative sinus rhythm.

Coordinates of the curve:		
Test result variable(s): %EF		
Positive if greater than or equal to^a^	Sensitivity	1 - Specificity
48.5000 (cutoff value)	0.787	0.506

The test result variable(s)=EF has at least one tie between the positive actual state group and the negative actual state group.

a The smallest cutoff value is the minimum observed test value minus 1, and the largest cutoff value is the maximum observed test value plus 1. All the other cutoff values are the averages of two consecutively ordered observed test values.

The ECGs of 61 (76.25%) out of 80 patients displayed SR during their course in the early postoperative period; whereas five (6.25%) of the patients remained in AF. Fourteen (17.5%) out of 80 patients required temporary pacemakers and permanent pacemakers were implanted in six (7.5%) of them (the implantations were performed in the early period in four patients and in the late period in two patients).

The ECGs of 61 (76.25%) out of 80 patients displayed SR in the first month of the postoperative period. Conversion to AF occurred in three more (72.5%) patients in the 6^th^ month of the postoperative period. The factors impacting on the conversion to SR in the early postoperative period and on its maintenance were assessed in patients with AF who underwent MVS and with adjunctive Cox-Maze III procedure by cryoablation. On the other hand, when patients were grouped as the ones with LAD ≤ 45 mm and patients with LAD ≥ 46 mm, the rates of remaining in SR were in favor of the latter group on the day of discharge and in the first postoperative month (*P*<0.004, *P*<0.026).

No statistically significant results were detected with ROC analysis when patients were classified in terms of the duration of CPB and cross-clamp period. There were no differences between the groups when the patients were classified according to gender.

## DISCUSSION

Isolation of PV from the atrial tissue treats AF in 65-80% of AF patients as current studies have shown that the largest ectopic foci initiating AF are the PV. In addition to the PV, other cardiac structures may be the ectopic foci at a rate of 20%, including the superior vena cava, free wall of the left atrium, terminal crest, coronary sinus ostium, Marshall ligament, and interatrial septum. In chronic AF, ectopic foci may occur at a rate of 35%^[12]^.

The pioneers of the curative ablation in AF were cardiac surgeons. Cox's Maze-III procedure started to be used in 1992 and has evolved by the compiling surgical experiences built by the collection of worldwide mapping studies conducted in animals and humans^[13]^. The lesions are created by the cut-and-sew technique, following a median sternotomy. Cox reported a permanent SR with this technique at a rate of 97%, which was found to be 84.9% in 2004, by a 1553-patient review^[14]^. The large patient series reported 30-day mortality rates between 0-7.2% and a stroke rate of 0.5%, as well as the requirement for permanent pacemakers at a rate of 5.8% and bleeding due to multiple incisions at a rate of 4.9%, therefore, the application of the procedure has been limited due to these high mortality and morbidity rates for this somewhat benign arrhythmia^[14,15]^. The Cox-Maze III procedure was applied in this study with cryoablation as a relatively new technique. In this present study, a total of 80 patients with mitral valve disease who underwent cryoablation with Cox-Maze-III procedure were included, and predefined patient factors which might potentially affect the conversion to SR were evaluated. When the preoperative LAD values of the patients were tied to the “sinus rhythm on the day of discharge” variable in the ROC analysis, a cutoff value of 44.5 mm was found. When the preoperative LAD values were tied to the “sinus rhythm in the first month” variable, a cutoff value of 45.5 mm was found. No ties were made with other parameters. Based on the results of the Chi-square test, the patients with LAD ≥ 46 mm were found to achieve higher rates of conversion to SR on the day of discharge and in the first month compared to the patients with LAD ≤ 45 mm. Many clinical investigators have set thresholds of the LAD in order to predict the efficacy of surgical treatment of AF. Melo et al.^[16]^ reported the boundary of 5.5 cm and Yin et al.^[17]^ set the threshold at 5.8 cm and Cao et al.^[18]^ at 6.8 cm. In 2008, Breda Jr. et al.^[19]^ published a similar study with small amount of patients using radiofrequency for ablation, reporting that two patients with LAD 65 and 68 mm presented with relapses of AF rhythm after discharge.

A relatively smaller LAD is a more effective factor in determining the conversion to the SR after the surgical ablation in AF patients; however, our study results showed that LAD values ≥ 46 mm were more favorable in the early postoperative period and in the first month. Although previous studies in the literature found out that smaller LAD were more favorable for surgical ablation, the cutoff value of 45.5 mm found in our study, which was smaller than the reported values by the previous studies in the literature, suggested that LAD was not the only factor determining the conversion to the SR.

For the preoperative EF values, a cutoff value of 48.5% was found based on the ROC analysis’ results. A higher value of EF (%) in the early postoperative period was found to be a significant factor in determining the conversion to the SR. A similar result was also obtained when an empirically attributed value of 45% was used in the analysis. However, all of these significant differences favoring the respective groups of patients could not be observed in the six-month follow-up visits. In the ROC analysis, no significant differences were observed in other variables including gender, total duration of CPB, or duration of cross-clamping.

In the six-month follow-up visits, 14 patients (17.5%) required transient and six patients (7.5%) required permanent pacemakers. Of these six patients, four required the permanent pacemaker in the early period and the remaining two patients required it in the late period. Since this study is limited to 80 patients without early postoperative mortality, larger randomized studies are warranted to evaluate the benefits to be achieved by the application of this actually controversial procedure in the long term, as 7.5% of the patients required permanent pacemakers in this study. In this procedure, the increased requirement for the placement of temporary and permanent pacemakers is the most criticizing event.

Performance of the Maze procedure adjunctive to the mechanical valve replacement is still debated as it is still uncertain whether it decreases the risk of TE events alone when compared to the anticoagulant treatment. It hinders the recovery of cardiac function in the early postoperative period by increasing the duration of the CPB procedure and the cardiac ischemia. In addition, it increases the frequency of bradyarrhythmias^[20,21]^. A 5466-patient review conducted in 2005 followed up the patients for an average duration of 6.6 years and reported that conversion to SR after the valvular surgery did not increase the survival or decrease the number of TE events^[22]^. No mortality occurred in our study as well; however, 14 patients required temporary and six patients required permanent pacemakers. In 2008, Gomes Junior et al.^[23]^ published a similar study on 33 patients undergoing ablation with electrocautery for AF during MVS. The authors presented parallel results to ours and reported low mortality and morbidity rates. In our study, TE events occurred only in three patients in the early postoperative period and no further TE events occurred in the six-month follow-up period. Vural et al.^[24]^, in their recently published article (2018), have presented a comparison of cryoablation and radiofrequency ablation in MVS. They reported that there was no difference between the energy sources used for conversion to SR from AF. And they reported also that the rate of TE events was low (2.3%). These data suggested us that SR might be effective as much as the anticoagulant therapy for protection from TE events. Bagge et al.^[25]^ compared MVS alone to MVS in combination with cryoablation. They reported that left atrial cryoablation during MVS did not improve the health-related quality of life in patients with permanent AF.

## CONCLUSION

In this study, we discussed some factors affecting the conversion to SR during the postoperative period in patients with AF who underwent MVS with adjunct Cox-Maze III procedure by cryoablation. The favorably significant differences in conversion to SR were obtained on the day of discharge and at the postoperative follow-up visit only in the group of patients with LAD ≥ 45.5 mm. At the same time, the rates of conversion to SR during the early postoperative period were statistically higher in patients with high left ventricular EF values than in those with low EF values. However, these differences disappeared at the sixth-month follow-up visits. Of the patients, 17.5% required temporary and 7.5% required permanent pacemakers. This finding is one of the important aspects of this procedure to be criticized. Conversion to SR and its maintenance during the postoperative period will affect the cardiac functions and prevention of the TE events in the early period favorably. Performing cryoablation in adjunct with MVS in patients with AF leads to a longer duration of the surgical procedure, however, we are of the opinion that it may not affect mortality and morbidity during the short and moderate terms. In addition, we believe that the experience of the staff at the clinic, where the procedure is performed, and the devices used during the procedure are important factors too. It is observed that, in these patients, the benefits provided by the Cox-Maze III procedure may be as effective as those of anticoagulation treatment.

Considering the publications in the literature and the results of our study, it is clearly evident that comprehensive studies and extensive meta-analyses are required, comparing the cases that were applied Cox-Maze III procedure to the ones who were not.

**Table:** 

Authors' roles & responsibilities	
FST	Substantial contributions to the conception or design of the work; or the acquisition, analysis, or interpretation of data for the work; drafting the work or revising it critically for important intellectual content; agreement to be accountable for all aspects of the work in ensuring that questions related to the accuracy or integrity of any part of the work are appropriately investigated and resolved; final approval of the version to be published
MBE	Substantial contributions to the conception or design of the work; or the acquisition, analysis, or interpretation of data for the work; drafting the work or revising it critically for important intellectual content; agreement to be accountable for all aspects of the work in ensuring that questions related to the accuracy or integrity of any part of the work are appropriately investigated and resolved; final approval of the version to be published
AD	The acquisition, analysis, or interpretation of data for the work; final approval of the version to be published
